# Stomach position evaluated using computed tomography is related to successful post-pyloric enteral feeding tube placement in critically ill patients: a retrospective observational study

**DOI:** 10.1186/s40560-023-00673-4

**Published:** 2023-05-30

**Authors:** Masashi Yokose, Shunsuke Takaki, Yusuke Saigusa, Takahiro Mihara, Yoshinobu Ishiwata, Shingo Kato, Keiichi Horie, Takahisa Goto

**Affiliations:** 1 Department of Anesthesiology and Critical Care Medicine, Yokohama City University Graduate School of Medicine, 3-9 Fukuura, Kanazawa-Ku, Yokohama, Japan; 2 Department of Biostatistics, Yokohama City University Graduate School of Medicine, Yokohama, Japan; 3 Department of Health Data Science, Yokohama City University Graduate School of Data Science, Yokohama, Japan; 4 Department of Diagnostic Radiology, Yokohama City University Graduate School of Medicine, Yokohama, Japan

**Keywords:** Enteral nutrition, Post-pyloric feeding, Intensive care unit, Greater curvature of stomach, Computed tomography

## Abstract

**Background:**

Post-pyloric enteral feeding reduces respiratory complications and shortens the duration of mechanical ventilation. Blind placement of post-pyloric enteral feeding tubes (EFT) in patients with critical illnesses is often the first-line method because endoscopy or fluoroscopy cannot be easily performed at bedside; however, difficult placements regularly occur. We reported an association between the stomach position caudal to spinal level L1–L2, evaluated by abdominal radiographs after placement, and difficult placement; however, this method could not indicate difficulty before EFT placement. The aim of our study was to evaluate the association between stomach position, estimated using computed tomography (CT) images taken before the blind placement of the post-pyloric EFT, and the difficulty of EFT placement.

**Methods:**

Data from patients aged ≥ 20 years who underwent post-pyloric EFT in our intensive care unit were obtained retrospectively. Logistic regression analysis was used to evaluate the association between successful initial EFT placement and explanatory variables, including stomach position estimated by CT. Two cut-off values were used: caudal to L1–L2 based on a previous study and the best cut-off value calculated by the receiver operating characteristic curve. Variable selection was performed backward stepwise using Akaike's Information Criterion.

**Results:**

Of the total of 453 patients who were enrolled, the success rate of the initial EFT placement was 43.5%. The adjusted odds ratio for successful initial EFT placement of the stomach position caudal to L1–L2 was 0.61 (95% confidence interval: 0.41–1.07). Logistic regression analysis, including the stomach position caudal to L2–L3, calculated as the best cut-off value, indicated that stomach position was an independent factor for failure of initial EFT placement (adjusted odds ratio, 0.55; 95% confidence interval: 0.33–0.91).

**Conclusions:**

Stomach position evaluated using CT images was associated with successful initial post-pyloric EFT placement. The best cut-off value of the greater curvature of the stomach to predict the success or failure of the first attempt was spinal level L2–L3.

*Trial registration* University Hospital Medical Information Network Clinical Trials Registry (UMIN000046986; February 28, 2022). https://center6.umin.ac.jp/cgi-open-bin/ctr/ctr_view.cgi?recptno=R000052151

**Supplementary Information:**

The online version contains supplementary material available at 10.1186/s40560-023-00673-4.

## Background

Patients with critical illness are at high risk of moderate to severe malnutrition [1, 2]. Malnutrition in the intensive care unit (ICU) is associated with increased length of ICU stay, ventilator dependency, infection, and mortality [3]. International guidelines for nutrition in patients with critical illness recommend early initiation of enteral feeding within 24–48 h of ICU admission [4–6]. Enteral feeding is primarily provided through the stomach or post-pyloric route in critically ill patients. Compared to trans-gastric feeding, post-pyloric feeding reduces the risk of respiratory complications [7, 8] because of reduced gastric residual volume and potentially increased ease in achieving energy targets to avoid procedure interruptions. Furthermore, post-pyloric feeding is associated with a shorter duration of mechanical ventilation [9], and it can be suitable for patients who cannot elevate the head of their beds or have gastrointestinal intolerance during transgastric feeding.

In the ICU, blind placement, which relies on auscultation and resistance when advancing the enteral feeding tube (EFT) was commonly performed at the bedside. The reasons are as follows: (1) the viability of adapting support technologies like fluoroscopy and endoscopy was not practical for all patients with unstable respiratory or circulatory states who could not easily be transferred to other departments; (2) the department or specialists operating the assistive device may not always be immediately available; (3) not all hospitals have access to non-invasive special equipment such as electromagnetic [10] or ultrasound guidance [11] to support successful bedside placement. However, patients in the ICU often encounter difficulty in blind placement of post-pyloric EFT at the bedside, which may cause a delay in enteral feeding. We reported an association between the position of the greater curvature of the stomach estimated by abdominal radiographs to confirm the location of the EFT tip after the procedure and the difficulty of EFT placement [12]. However, radiography cannot be used to recognize difficulty before EFT placement. As a potential solution, computed tomography (CT) has a wide range of applications, including screening, diagnosis, evaluation of disease progression, and determination of treatment effectiveness. CT is likely to be performed before ICU admission because of its use, which frequently includes critically ill medical and post-major surgical patients. The ability to evaluate the difficulties of post-pyloric EFT placement using CT scans taken before ICU admission would be clinically beneficial.

This retrospective observational study aimed to evaluate the association between the position of the greater curvature of the stomach estimated by CT images and the difficulty of blind placement of the post-pyloric EFT.

## Methods

The study was approved by the Ethics Board of Yokohama City University Hospital, Yokohama, Japan (approval number: B201200065; December 28, 2020). The study was conducted in the University Medical Information Network Clinical Trials Registry (UMIN000046986; February 28, 2022; principal investigator, Masashi Yokose) before data acquisition. This study was performed in accordance with the principles of the Declaration of Helsinki. The requirement for informed written consent was waived due to the retrospective design of this study. The inclusion criteria included: (1) consecutive patients aged ≥ 20 years with post-pyloric EFT blindly placed after admission to the ICU of Yokohama City University Hospital between January 1, 2012, and November 30, 2020, and (2) patients who had CT scan data of the abdomen in the year before the EFT placement. Many of the participants in this study were consistent with those in our previous publication [12]. The exclusion criteria ruled out patients who had an EFT on admission to ICU, gastrostomy or enteric fistula, and previous upper gastrointestinal tract surgery.

### Protocol for blind placement of enteral feeding tube

In our institution, post-pyloric enteral nutrition is used as the first choice. All patients underwent EFT with a stylet (Kangaroo™ New Enteral Feeding Tube; Covidien Japan, Tokyo, Japan) through the nose or mouth. The size of the EFT (8–12 French) was selected to be suitable for the physique of the patient. Prokinetic agents were used when deemed clinically necessary by the physician performing the placement. The right lateral decubitus position was recommended. This was allowed at the discretion of the practicing physician or depending on the patient’s condition. First, the EFT tip was advanced to the stomach (40–65 cm) and checked by auscultation of air injection at the pit of the stomach. The EFT was then advanced several centimeters at one time. The advancement of the EFT in the stomach was verified by ensuring that the tube did not return to each advance. If the EFT had not advanced, it was pulled out by ~ 5 cm and then readvanced. The tip location of the EFT was estimated by auscultation of the air injected through the tube. If a high-pitched tone was heard on the right lateral side of the abdomen after advancing the EFT tip 20–30 cm from the point at which the EFT tip was confirmed to be in the stomach, 20 mL of air or water was administered. After confirming that none of the contents could be aspirated from the nasogastric tube, radiographs of the abdomen were obtained to check the position of the tip. This standardized procedure was a minor modification of a previously published protocol [13]. The position of the EFT tip was evaluated by a team that included at least one physician with extensive experience in post-pyloric placement, as described in the electronic clinical record. The decision to begin using the placed EFT was made by a consensus among several physicians.

### Data acquisition

The following variables for logistic regression analysis were collected from electronic medical records: (1) age; (2) sex; (3) height; (4) body mass index (BMI) at ICU admission; (5) patient category (i.e., medical or post-surgical admission); (6) Sequential Organ Failure Assessment score; (7) intestinal peristaltic movement; (8) use of prokinetic agents; (9) position of the stomach defined as the lowest point of the stomach relative to spinal level and as evaluated by the most recent CT of the abdomen within 1 year before EFT placement; (10) hiatal hernia; (11) diabetes mellitus; (12) body position during the procedure (i.e., right lateral or other positions); (13) experience of the physicians who perform the EFT placement (i.e., junior/senior ICU residents or others); (14) use of renal replacement therapy; (15) fluid balance defined as weight change between ICU admission and first EFT placement; (16) serum albumin levels, (17) use of sedatives; (18) use of opioids; (19) use of vasopressor agents; (20) use of cardiac assist devices (extracorporeal membrane oxygenation, intra-aortic balloon pumping, or ventricular assist device). Serum albumin levels and Sequential Organ Failure Assessment scores were collected from the data nearest to EFT placement. The position of the stomach was read by radiologists who did not evaluate the success or failure of EFT placement and were independent of the data analysis. The position of the stomach was handled as binary, either cephalad or caudal to spinal level L1–L2, based on our previous study [12], and was defined as the provisional cut-off value. Enteral feeding-related outcomes (i.e., days between admission to ICU and start enteral nutrition and days between EFT placement and starting enteral nutrition, ventilator-free days (VFDs), ICU length of stay, and deaths in the hospital) were obtained from medical records. The definition of VFDs was 28 days minus the number of days of mechanical ventilation with endotracheal intubation. The information on the EFT procedure was as follows: (1) the number of procedures required for successful post-pyloric EFT placement; (2) the number of participants who underwent gastrointestinal fibroscopy or fluoroscopy; (3) the number of participants who abandoned post-pyloric placement; (4) the position of the greater curvature at the first successful post-pyloric placement.

### Outcomes

**Fig. 1:**
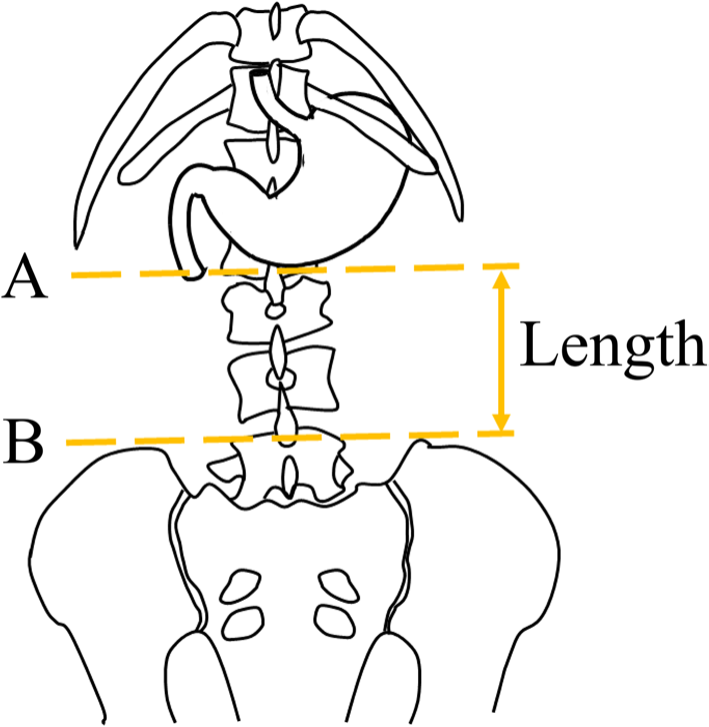
The definition of the length between the stomach and iliac crest. The dashed line A is the lowest point of the greater curvature of the stomach. The dashed line B is the line of the superior border of the iliac crest. The length was defined as the vertical distance between lines A and B

**Fig. 2:**
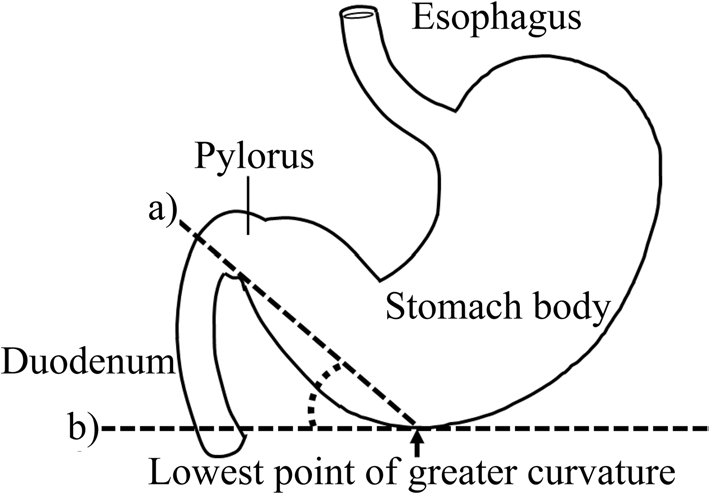
The definition of the angle. Line (a) connects the lowest point of the serosal side of the greater curvature of the stomach to the lower point of the caudal and serosal side of the pylorus. Line (b) indicates the horizontal line

The primary outcome of this study was the rate of successful initial post-pyloric EFT placement. Three experienced physicians independently determined the first-pass success using abdominal radiography after the first attempt. In cases of disagreement, a comprehensive decision was made based on discussions by the three researchers. The secondary outcomes were as follows: (1) the association between the number of attempts required for successful post-pyloric EFT placement and stomach position estimated by CT; (2) the correlation coefficient between the vertical length from the line of the superior border of the iliac crest to the lowest point of the greater curvature of the stomach (Fig. 1) and stomach position estimated by CT; (3) the correlation coefficient between the angle calculated by (a) the line between the lowest point of the serosal side of the greater curvature and the lower point of the caudal and serosal side of the pylorus, and (b) the horizontal line (Fig. 2) and stomach position estimated by CT; (4) a description of patient outcomes and information on the EFT procedure.

### Sample size calculation

A prior statistical sample size calculation was not performed due to the retrospective nature of this research. The maximum number of patients that could be obtained during the study period was defined as the sample size.

### Statistical analyses

All data are presented as numbers (percentages) or medians (interquartile range), as appropriate. In the univariate analysis for patient characteristics between the success and failure groups, the unpaired t-test, Mann–Whitney U test, or Fisher’s exact test were appropriately conducted. The association between the success of the first placement and the explanatory variables was evaluated using logistic regression analysis. Variable selection was performed backward stepwise as guided by Akaike’s Information Criterion [14]. Age, sex, BMI, and stomach position estimated using CT were forcibly entered into the final model. Multicollinearity between all the predictors was checked using the variance inflation factor. Missing values were incomplete or summarized for each factor. The determination of the best cut-off point of stomach position estimated by CT for predicting the success of the first placement was performed using a receiver operating characteristic (ROC) curve. The cut-off value was determined based on Youden’s index [15]. Multiple regression analysis was used to determine the factors associated with the number of attempts until successful blind EFT placement. Variable selection and forced entry variables were performed using the same process as that for the analysis of the primary outcome. Spearman’s rank coefficients were calculated to assess the correlation coefficients of the secondary outcomes. Statistical significance was set at a P value < 0.05 (two-tailed). All statistical analyses were performed using R software (version 4.2.1: the R Foundation for Statistical Computing; Vienna, Austria), JMP Pro software ver. 15.0 (SAS Institute; Cary, NC, US), and Microsoft Excel 2021 (Microsoft, Redmond, WA, US).

## Results

**Fig. 3:**
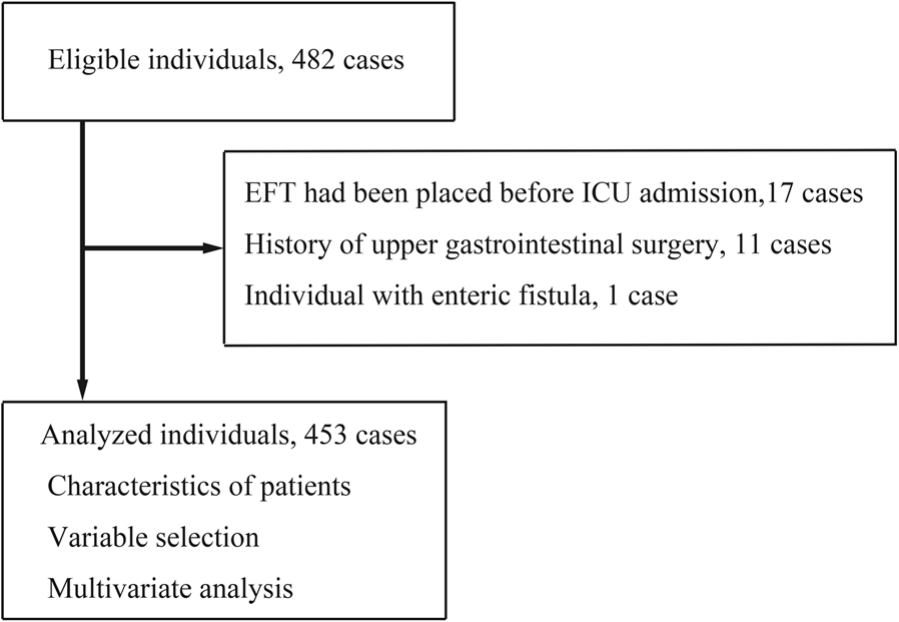
Study flowchart. *EFT* enteral feeding tube, *ICU* intensive care unit

Of the 534 individuals screened before data collection, 482 were enrolled with available CT images. After excluding 17 patients who underwent EFT before ICU admission, 11 with previous upper gastrointestinal tract surgery, and 1 with enteric fistula, 453 were included in the analysis (Fig. 3). CT images to evaluate stomach position were taken 3 (1–17) days before EFT placement. The median values of age, height, and BMI were 68 (57–76) years, 162 (155–168) cm, and 22.5 (19.9–25.4) kg/m^2^, respectively (Table 1). The number of patients requiring postoperative management was slightly higher than that of medical patients (Table 1). The median number of days in the ICU was 8 (6–15), and the median VFDs were 20 (6–24) days. Thirty-four patients (7%) died in the ICU, and 141 (31%) died in the hospital. The number of days between admission to the ICU and the start of enteral nutrition was 2 (1–4) (Table 2).

**Table 1:** Characteristics of participants

	Overall (*n* = 453)	Success (*n* = 197)	Failure (*n* = 256)	*P*-value
Age (years)	68 (57–76)	68 (53–75)	69 (60–77)	0.07
Male sex, *n* (%)	272 (60)	107 (24)	165 (36)	0.03
Height (cm)	162 (155–168)	161 (155–168)	162 (155–168)	0.69
Missing value, *n* (%)	14 (3)	5 (1)	9 (2)	
Weight (kg)	59 (49–68)	60 (50–69)	58 (49–66)	0.31
Body mass index (kg/m^2^)	22.5 (19.9–25.4)	22.9 (20.1–25.7)	22.4 (19.8–25.0)	0.16
Missing value, *n* (%)	14 (3)	5 (1)	9 (2)	
Patient category				
Medical, *n* (%)	209 (46)	98 (22)	111 (24)	0.18
Surgical, *n* (%)	244 (54)	99 (22)	145 (32)	
SOFA score at initial procedure	11 (8–13)	10 (8–13)	11 (8–13)	0.41
Absence of intestinal peristalsis, n (%)	357 (79)	151 (34)	205 (45)	0.42
Use of a prokinetic agent, n (%)	28 (6)	15 (3)	13 (3)	0.33
Position of the stomach evaluated by CT	L2 (L1 to L3)	L2 (L1 to L3)	L2 (L1 to L3)	0.01
> L2, n (%)	172 (38)	83 (18)	89 (20)	0.12
≤ L2, n (%)	281 (62)	114 (25)	167 (37)	
Length (cm)	9.2 (6.3–12.4)	10.0 (6.9–13.2)	8.7 (6.1–11.7)	0.002
Missing value, *n* (%)	16 (4)	6 (1)	10 (2)	
Angle (°)	21 (0–35)	20.0 (0–32.0)	21.1 (0–36.7)	0.17
Hiatal hernia, *n* (%)	21 (5)	8 (2)	13 (3)	0.66
Diabetes mellitus, n (%)	95 (21)	43 (10)	52 (11)	0.73
Right lateral position, n (%)	190 (42)	78 (17)	112 (25)	0.39
Degree of experience				
Junior/senior resident, *n* (%)	105 (23)	51 (12)	54 (11)	0.16
Others, *n* (%)	260 (57)	105 (23)	155 (34)	
Missing values, *n* (%)	88 (19)	41 (9)	47 (10)	
Renal replacement therapy, *n* (%)	59 (13)	21 (5)	38 (8)	0.21
Fluid balance from baseline (kg)	2.2 (0.3–4.7)	2.2 (0.0–4.9)	2.2 (0.7–4.6)	0.82
Serum albumin (g/dL)	2.7 (2.3–3.1)	2.7 (2.3–3.1)	2.7 (2.3–3.1)	0.97
Use of sedatives, *n* (%)	370 (82)	152 (34)	218 (48)	0.04
Fentanyl dose (µg/h)	20 (10–20)	20 (0–20)	20 (10–26.3)	0.07
Use of a vasopressor, *n* (%)	331 (73)	136 (30)	195 (43)	0.11
Use of cardiac assist devices, *n* (%)	40 (11)	17 (5)	23 (5)	1.00

*Angle* was calculated by (a) the line between the lowest point of the serosal side of the greater curvature of the stomach and the lower point of the caudal and serosal sides of the pylorus and (b) the horizontal line. *Body mass index* was calculated based on height and weight at ICU admission. *Cardiac assist devices* included extracorporeal membrane oxygenation, ventricular assist devices, and intra-aortic balloon pumping. *Fluid balance from baseline* was defined as an increase or decrease in body weight from ICU admission to the first EFT placement. *Length* was defined as the vertical length from the line of the superior border of the iliac crest to the lowest point of the greater curvature of the stomach. *Success* was the first successful attempt at post-pyloric EFT placement. *Failure* was the first attempt at failure for post-pyloric EFT placement. All values were expressed as numbers (percentages) or medians (interquartile ranges)

*CT* computed tomography, *EFT* enteral feeding tube, *ICU* intensive care unit, *SOFA* Sequential Organ Failure Assessment

**Table 2:** Outcome data of participants

	Overall (*n* = 453)	Success (*n* = 197)	Failure (*n* = 256)
Length of ICU stay (days)	8 (6–15)	9 (6–14)	8 (6–15)
Died in the ICU, *n* (%)	34 (7)	17 (4)	17 (4)
Died in the hospital, *n* (%)	141 (31)	61 (13)	80 (18)
Died after ICU admission (days)	26 (14–53)	30 (15–54)	26 (14–51)
Ventilator-free days (days)	20 (6–24)	21 (8–24)	20 (5–23)
Time between EFT placement and the start of EF (days)	1 (0–2)	0 (0–1)	1 (1–2)
Time between ICU admission and the start of EF (days)	2 (1–4)	2 (1–3)	2 (2–4)
Patients in which enteral nutrition could not be performed, *n* (%)	18 (4)	4 (1)	14 (3)

*Success* was the first successful attempt at post-pyloric EFT placement. *Failure* was the first attempt at failure for post-pyloric EFT placement. *Ventilator-free days* were calculated as 28 days minus the days of mechanical ventilation with endotracheal intubation. All values are expressed as median (interquartile range) or number (percentage). *EF* enteral feeding, *EFT* enteral feeding tube, *ICU* intensive care unit.

Successful post-pyloric EFT placement was eventually achieved in 328 (72.4%) patients (Table 3). The success rate for the initial post-pyloric EFT placement was 43.5% (*n* = 197). The success rate for the second and subsequent attempts was 28.9% (*n* = 131). The number of patients who underwent gastrointestinal fibroscopy or fluoroscopy was 13 (2.9%).

**Table 3:** The data on the procedure of enteral feeding tube

No of patients who succeeded post-pyloric EFT placement, *n* (%)	328 (72.4)
First attempt success, *n* (%)	197 (43.5)
Success in second and subsequent attempts, *n* (%)	131 (28.9)
Number of patients who succeeded in ≤ 3 attempts, *n* (%)	109 (24.1)
Number of patients who succeeded in ≥ 4 attempts, *n* (%)	22 (4.9)
Number of attempts to success with blind placement after second attempt	2 (2–3)
Lowest position of EFT in stomach	
Success in first attempt	L1 (Th12–L2)
Success in second and subsequent attempt	L2 (L1–L3)
Number of patients with failure to place EFT in the post pylorus, *n* (%)	112 (24.7)
Number of patients using the assist device, *n* (%)	13 (2.9)
Gastro-intestinal fiberscope, *n*	10
Fluoroscopy, *n*	3
Number of attempts before using assist devices	2 (2–4)
Length (cm)	
Stomach position > L2	12.8 (11.2–14.8)
Stomach position ≤ L2	7.3 (4.9–9.4)
Correlation, length and stomach position estimated by CT	0.84 (95% CI: 0.81–0.87)
Angle (°)	
Stomach position > L2	6.0 (− 13.9–20.3)
Stomach position ≤ L2	28.4 (15–40.0)
Correlation, angle and stomach position estimated by CT	0.56 (95% CI 0.49–0.62)

*Angle* was calculated by (a) the line between the lowest point of the serosal side of the greater curvature of the stomach and the lower point of the caudal and serosal sides of the pylorus and (b) the horizontal line. *Length* was defined as the vertical length from the line of the superior border of the iliac crest to the lowest point of the greater curvature. *Stomach position* was the lowest position of the greater curvature, as estimated from the CT images. All values are expressed as median (interquartile range) or number (percentage)

*CI* confidence interval, *CT* computed tomography, *EFT* enteral feeding tube

The assessment of multicollinearity before the logistic regression analysis indicated that the risk was low because of the low variance inflation factor. The details of the logistic regression analysis, including all variables, are shown in Additional file 1. After variable selection from 20 variables, 8 explanatory variables (age, BMI, sex, patient category, experience of physician, position of the stomach, use of vasopressors, and use of opioids) were selected for the final analysis (Table 4). The stomach position caudal to L1–L2 tended to be associated with unsuccessful placement of the EFT; however, this was not statistically significant [adjusted odds ratio, 0.61; 95% confidence interval (CI) 0.41–1.07; *P* = 0.09]. Non-resident physicians (adjusted odds ratio, 0.61; 95% CI 0.37–0.99; *P* = 0.046) and use of opioids (adjusted odds ratio, 0.54; 95% CI 0.31–0.94; *P* = 0.03) were statistically significant factors for the failure of initial placement. The ROC curve demonstrated the best cut-off value for stomach position estimated by CT for predicting the failure of the first placement was caudal to L2–L3 (see Additional file 2). Table 4 also shows the logistic regression model, including the stomach position caudal to L2–L3. Eight variables were selected after variable selection, similar to the analysis using the stomach position caudal to L1–L2. The adjusted odds ratio of the stomach position caudal to L2–L3 for first-pass success was 0.55 (95% CI 0.33–0.91; *P* = 0.02). The use of opioids was also a significant factor (adjusted odds ratio, 0.57; 95% CI 0.33–0.99, *P* = 0.049).

**Table 4:** Results of multivariate analysis for primary outcome

Variables	Odds ratio	95% CI	*P*-value
Provisional cut-off: cephalad or caudal to L1–L2			
Age (10-year increments)	0.90	0.76–1.05	0.17
Body mass index	1.03	0.98–1.09	0.20
Sex (female)	1.40	0.88–2.25	0.16
Patient category (surgical)	0.73	0.47–1.15	0.17
Experience of physician (non-resident)	0.61	0.37–0.99	0.046
Position of the stomach (caudal to L1–L2)	0.61	0.41–1.07	0.09
Use of vasopressors	0.70	0.43–1.15	0.16
Use of opioids	0.54	0.31–0.94	0.03
Best cut-off value: cephalad or caudal to L2–L3			
Age (10-year increments)	0.90	0.77–1.05	0.18
Body mass index	1.03	0.98–1.09	0.27
Sex (female)	1.48	0.92–2.38	0.11
Patient category (surgical)	0.72	0.46–1.14	0.17
Experience of physician (non-resident)	0.63	0.38–1.03	0.07
Position of the stomach (caudal to L2–L3)	0.55	0.33–0.91	0.02
Use of vasopressors	0.68	0.41–1.11	0.12
Use of opioids	0.57	0.33–0.99	0.049

Odds ratio > 1.0 are associated with successful placement of enteral feeding tubes. *CI* confidence interval

Multiple regression analysis using the data of 328 patients with successful post-pyloric EFT placement at first attempt or after multiple attempts revealed that the position of the stomach caudal to L2–L3; as well as older age; male sex; low BMI; use of sedatives or vasopressors; circulation assistance at first placement; no diabetes mellitus; and hiatal hernia increased the total number of attempts until successful EFT placement; however, these were not statistically significant (Table 5).

**Table 5:** Multiple regression analysis for total attempt number until successful EFT placement

Variables	Coefficient (95% CI)	*P*-value
Age (10-year increments)	0.053 (− 0.028–0.133)	0.20
Sex (female)	− 0.173 (− 0.419–0.073)	0.17
BMI (1-point increments)	− 0.008 (− 0.035–0.018)	0.55
Use of sedatives at first attempt	0.208 (− 0.088–0.504)	0.20
Use of vasopressors at first attempt	0.245 (− 0.021–0.511)	0.12
Use of cardiac assist devices at first attempt	0.321 (− 0.060–0.702)	0.10
Diabetes mellitus	− 0.258 (− 0.543–0.027)	0.08
Position of the stomach (caudal to L2–L3)	0.108 (− 0.162–0.379)	0.43
Hiatal hernia	0.329 (− 0.209–0.867)	0.23

This analysis was performed using the data of 328 patients with successful post-pyloric EFT placement at first attempt or after multiple attempts. *Cardiac assist devices* include extracorporeal membrane oxygenation, intra-aortic balloon pumping, or ventricular assist devices

*BMI* body mass index, *CI* confidence interval, *EFT* enteral feeding tube

The length between the iliac horizontal line and the lowest point of the greater curvature estimated by CT was significantly longer in patients with first-pass success (10.0 cm; 95% CI 6.9–13.2 cm) than in those with failure (8.7 cm; 95% CI 6.1–11.7 cm) (Table 1). The correlation between the length from the iliac horizontal line to the lowest point of the stomach and stomach position evaluated by CT was 0.84; 95% CI 0.81–0.87 (Table 3). The correlation coefficient between the angle and stomach position evaluated by CT was 0.56; 95% CI 0.49–0.62 (Table 3).

## Discussion

Our results revealed that the stomach position evaluated by CT obtained before the initial procedure was associated with success or failure of the first placement of the post-pyloric EFT by the blind method. The best cut-off value for failure at first attempt of EFT placement, estimated using CT imaging, was the greater curvature lower than spinal level L2–L3.

A previous study [12] used abdominal radiographs after EFT placement to estimate the position of the stomach; however, this method was not clinically effective enough to recognize the difficulty before starting blind placement. Moreover, the position of the stomach estimated using abdominal radiography after EFT placement could have been modified by air injection or stretching of the stomach wall during the placement procedure. The strength of this study is that the effect of stomach position was evaluated using CT images taken before the EFT placement, and these images were not modified by the EFT placement procedure. We demonstrated the association between difficulty at the first attempt of EFT placement and lower stomach position, reaffirmed the hypothesis of a previous study [12].

The clinical application of our findings is influenced by the frequency with which available CT images are present at the time of EFT placement. At the time of screening before collecting data, approximately 90% of patients who underwent EFT had available CT images. Considering the large number of CT scanners per population in our country [16], it may be the lower frequency with available CT images at the EFT placement in our study than in other countries. However, the fact that this study showed an association between CT images and successful post-pyloric EFT placement even though it included CT images acquired well before placement (i.e., not just immediately before) demonstrates the value through wider potential application of our findings.

In patients in whom EFT placement was successfully achieved, the total number of attempts was not substantially influenced by the low stomach position in secondary outcomes (Table 5). Therefore, it remains controversial whether methods other than blind placement of the EFT should be performed on the first attempt when the low stomach position was detected before the first attempt. Of the 328 patients who eventually underwent successful post-pyloric EFT placement, 308 (93.3%) had successful outcomes by the third attempt (Table 3). This information may help make a clinical decision to apply other assistive methods such as fluoroscopy or gastroscopy.

We hypothesized that the length and angle could be alternative indicators of the lowest position of the greater curvature relative to the spinal level. The length and angle had a relatively high correlation with the greater curvature relative to the spine estimated by CT (Table 3). The post-hoc logistic regression analysis using length or angle instead of stomach position revealed that difficulty in post-pyloric EFT placement was associated with length but not angle (see Additional files 3 and 4). Assessing the lowest position of the greater curvature relative to the spinal level as estimated using CT images may be a slightly complicated procedure. Spinal deformities, such as scoliosis of the spine, compression fracture, or lumbosacral transitional vertebra [17], may affect the estimation of stomach position using the spinal level. Therefore, the length, which is easier to measure, may be a more practical and objective indicator for daily clinical use. The angle was more likely to predict success because the steep angle resulting from the caudal extension of the stomach by the EFT placement may be expected to contribute to the difficulty in guiding the EFT tip to the pylorus. However, the angle calculated from the stomach at the time of CT imaging may not have been able to reflect the change in the shape of the stomach due to the EFT placement.

In our study, physician inexperience was associated with the success of EFT placement. The reasons for this result, which was contrary to the generally expected effects, could be speculated as follows. First, supervisors in our institution might have been more responsible than residents for performing the EFT placement on patients with more serious illnesses. These patients often had risk factors for upper gastrointestinal hypomotility and were often admitted at night or on holidays when human resources were insufficient, making it difficult to provide adequate time for EFT placement. Residents tended to be assigned to patients whose general condition was relatively stable or when there was an available time for placement. Second, the nursing care record had a significant number of missing data related to physicians’ experiences. The exact effect of the physician’s experience could not be determined due to the retrospective nature of the study design. A sensitivity analysis of the primary outcome that did not include the physician’s experience as a covariate showed that the effect of stomach position was consistent (see Additional file 5).

Our study revealed that opioid use was associated with difficulty in EFT placement. Opioids are one of the factors that affect gastrointestinal function in critically ill patients. The effects of morphine on upper gastrointestinal motility, including enhanced relaxation of the proximal stomach, increased pyloric tone, and retrograde duodenal contractions [18–20]. In enteral nutrition, fentanyl was associated with increased volume of gastric aspiration and upper digestive intolerance [21]. Because opioids are major agents for pain management in the ICU setting and are only one of the many factors involved in gastrointestinal function, the clinical intervention that withholds using opioids solely for EFT placement cannot be easily implemented.

### Limitations

First, this single-center study was performed in an urban educational university hospital in Japan. Whether other countries or institutions can provide similar results is unknown. Furthermore, the effects of unidentified confounders were not taken into account because the study was retrospective. Second, the data of patients who did not undergo CT before ICU admission were not included. This may include selection bias regarding emergency patients who could not afford CT scans or patients with central nervous system disease, stroke, or cerebral hemorrhage, which might have been infrequent for abdominal CT scans. Third, our study could not collect data about the time required for EFT placement, which was one of the indicators of difficulty, because it was not mentioned in the medical records.

## Conclusions

The position of the stomach evaluated by CT before the initial blind placement was associated with successful initial post-pyloric EFT placement. The best cut-off value of the greater curvature of the stomach, evaluated by CT imaging, to predict the failure of the first attempt was caudal to spinal level L2–L3. Length and angle could be alternative indicators of the position of the greater curvature relative to the spine. In particular, the length may be a more practical and objective indicator for daily clinical use because it is easier to measure.

## Supplementary Information

**Additional file 1.** Results of logistic regression analysis using all variables. The logistic regression analysis including all variables before performing the variable selection.

**Additional file 2.** Receiver operating characteristic curve for stomach position estimated by computed tomography. The best cut-off value for the successful placement of the stomach position estimated by computed tomography before the first placement of enteral feeding tube.

**Additional file 3.** Results of logistic regression analysis using length. The post-hoc logistic regression analysis using length instead of stomach position.

**Additional file 4.** Results of logistic regression analysis using angle. The post-hoc logistic regression analysis using angle instead of stomach position.

**Additional file 5.** Sensitivity analysis for primary outcome excluding experience of the physician. Sensitivity analysis of the primary outcome that did not include physician experience as a covariate.

## Data Availability

The datasets used and/or analyzed during the current study are available from the corresponding author upon reasonable request.

## Abbreviations

BMI: Body mass index
CI: Confidence interval
CT: Computed tomography
EFT: Enteral feeding tube
ICU: Intensive care unit
VFDs: Ventilator-free days
