# Supplementary material for: Stomach position evaluated using computed tomography is related to successful post-pyloric enteral feeding tube placement in critically ill patients: a retrospective observational study
Source: J Intensive Care. 2023 May 30;11:25. doi: 10.1186/s40560-023-00673-4 (PMC10228095; doi:10.1186/s40560-023-00673-4)
Supplement: Supplementary file 1 — Additional file 1. Results of logistic regression analysis using all variables. The logistic regression analysis including all variables before performing the variable selection. [file 40560_2023_673_MOESM1_ESM.docx]

**Additional file 1.** Results of logistic regression analysis using all variables

For stomach position cephalad or caudal to L1-2

| Variables | Odds ratio | 95% CI | *P*-value |
| --- | --- | --- | --- |
| Age (each 10-year increment) | 0.91 | 0.77−1.08 | 0.30 |
| Sex (female) | 1.73 | 0.89−3.35 | 0.10 |
| Height (each 10-cm increment) | 1.25 | 0.91−1.73 | 0.17 |
| Body mass index | 1.04 | 0.99−1.10 | 0.14 |
| Patient category (surgical) | 0.64 | 0.37−1.11 | 0.11 |
| SOFA score (each 1-point increment) | 0.99 | 0.91−1.09 | 0.92 |
| Presence of intestinal peristaltic movement | 0.81 | 0.45−1.46 | 0.47 |
| Use of prokinetic agents | 0.95 | 0.38−2.41 | 0.92 |
| Position of the stomach (caudal to L1-2) | 0.68 | 0.41−1.13 | 0.13 |
| Hiatal hernia | 0.65 | 0.19−2.21 | 0.49 |
| Diabetes mellitus | 1.24 | 0.69−2.25 | 0.48 |
| Body position (right lateral position) | 0.81 | 0.51−1.29 | 0.37 |
| Experience of physician (non-resident) | 0.59 | 0.35−0.98 | 0.04 |
| Renal replacement therapy | 0.79 | 0.38−1.64 | 0.52 |
| Fluid balance (each 1-kg increment) | 1.05 | 0.99−1.13 | 0.13 |
| Serum albumin (each 1 mg/dL increment) | 1.12 | 0.72−1.74 | 0.61 |
| Use of sedatives | 0.63 | 0.32−1.22 | 0.17 |
| Use of opioid | 0.58 | 0.32−1.05 | 0.07 |
| Use of vasopressor agents | 0.76 | 0.41−1.41 | 0.39 |
| Cardiac assist devices | 1.12 | 0.47−2.69 | 0.79 |

Odds ratio >1.0 are associated with successful placement of enteral feeding tube. *CI* confidence interval, *SOFA score*, Sequential Organ Failure Assessment scores.

For stomach position cephalad or caudal to L2-3

| Variables | Odds ratio | 95% CI | *P*-value |
| --- | --- | --- | --- |
| Age (each 10-year increment) | 0.92 | 0.77−1.09 | 0.34 |
| Sex (female) | 1.86 | 0.95−3.65 | 0.07 |
| Height (each 10-cm increment) | 1.26 | 0.91−1.74 | 0.16 |
| Body mass index | 1.04 | 0.98−1.10 | 0.19 |
| Patient category (surgical) | 0.63 | 0.36−1.09 | 0.10 |
| SOFA score (each 1-point increment) | 0.99 | 0.91−1.09 | 0.90 |
| Presence of intestinal peristaltic movement | 0.80 | 0.44−1.45 | 0.46 |
| Use of prokinetic agents | 1.04 | 0.41−2.66 | 0.93 |
| Position of the stomach (caudal to L2-3) | 0.56 | 0.33−0.95 | 0.03 |
| Hiatal hernia | 0.65 | 0.19−2.18 | 0.49 |
| Diabetes mellitus | 1.25 | 0.70−2.26 | 0.45 |
| Body position (right lateral position) | 0.81 | 0.51−1.28 | 0.37 |
| Experience of physician (non-resident) | 0.61 | 0.37−1.03 | 0.06 |
| Renal replacement therapy | 0.81 | 0.39−1.69 | 0.58 |
| Fluid balance (each 1-kg increment) | 1.05 | 0.98−1.13 | 0.13 |
| Serum albumin (each 1 mg/dL increment) | 1.14 | 0.73−1.77 | 0.57 |
| Use of sedatives | 0.67 | 0.34−1.31 | 0.24 |
| Use of opioid | 0.60 | 0.33−1.10 | 0.10 |
| Use of vasopressor agents | 0.74 | 0.40−1.36 | 0.33 |
| Cardiac assist devices | 1.18 | 0.49−2.81 | 0.72 |

Odds ratio >1.0 are associated with successful placement of enteral feeding tube. *CI* confidence interval, *SOFA score*, Sequential Organ Failure Assessment scores.
