# Supplementary material for: Stomach position evaluated using computed tomography is related to successful post-pyloric enteral feeding tube placement in critically ill patients: a retrospective observational study
Source: J Intensive Care. 2023 May 30;11:25. doi: 10.1186/s40560-023-00673-4 (PMC10228095; doi:10.1186/s40560-023-00673-4)
Supplement: Supplementary file 2 — Additional file 2. Receiver operating characteristic curve for stomach position estimated by computed tomography. The best cut-off value for the successful placement of the stomach position estimated by computed tomography before the first placement of enteral feeding tube. [file 40560_2023_673_MOESM2_ESM.docx]

**Additional file 2.** Receiver operating characteristic curve for stomach position estimated by computed tomography


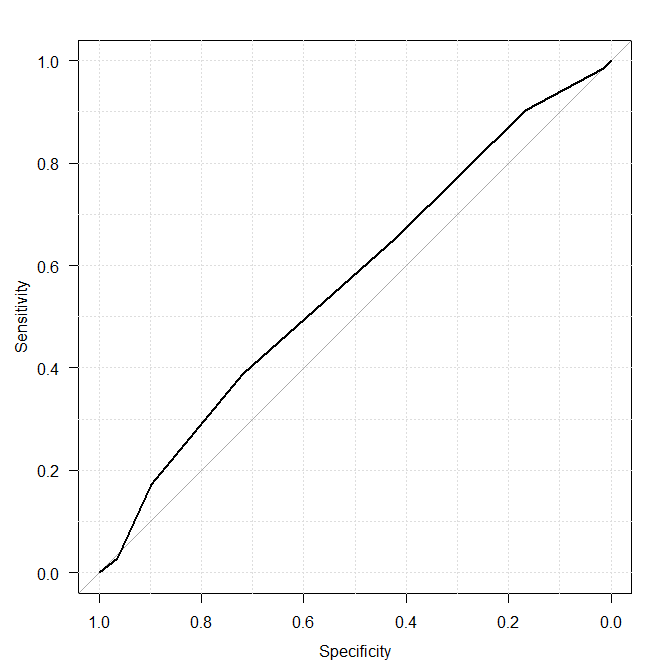


Best cut-off value for successful placement of stomach position estimated by computed tomography before first placement of enteral feeding tube was L2-3. Sensitivity was 0.72 and specificity was 0.39.
