# Supplementary material for: Stomach position evaluated using computed tomography is related to successful post-pyloric enteral feeding tube placement in critically ill patients: a retrospective observational study
Source: J Intensive Care. 2023 May 30;11:25. doi: 10.1186/s40560-023-00673-4 (PMC10228095; doi:10.1186/s40560-023-00673-4)
Supplement: Supplementary file 3 — Additional file 3. Results of logistic regression analysis using length. The post-hoc logistic regression analysis using length instead of stomach position. [file 40560_2023_673_MOESM3_ESM.docx]

**Additional file 3.** Results of logistic regression analysis using length

| Variables | Odds ratio | 95% CI | *P*-value |
| --- | --- | --- | --- |
| Age (each 10-year increment) | 0.93 | 0.79−1.09 | 0.39 |
| Body mass index | 1.03 | 0.98−1.09 | 0.25 |
| Sex (female) | 1.43 | 0.87−2.30 | 0.16 |
| Patient category (surgical) | 0.68 | 0.43−1.08 | 0.10 |
| Experience of physician (non-resident) | 0.62 | 0.37−1.03 | 0.06 |
| Length (each 10-cm increment) | 2.18 | 1.22−3.88 | 0.007 |
| Use of sedatives | 0.56 | 0.31−1.03 | 0.07 |
| Use of vasopressor | 0.66 | 0.40−1.09 | 0.10 |

*Length* was defined as the vertical length from the line of the superior border of the iliac crest to the lowest point of the greater curvature of stomach. Odds ratio >1.0 are associated with successful placement of enteral feeding tube. *CI* confidence interval.
