# Supplementary material for: Stomach position evaluated using computed tomography is related to successful post-pyloric enteral feeding tube placement in critically ill patients: a retrospective observational study
Source: J Intensive Care. 2023 May 30;11:25. doi: 10.1186/s40560-023-00673-4 (PMC10228095; doi:10.1186/s40560-023-00673-4)
Supplement: Supplementary file 4 — Additional file 4. Results of logistic regression analysis using angle. The post-hoc logistic regression analysis using angle instead of stomach position. [file 40560_2023_673_MOESM4_ESM.docx]

**Additional file 4.** Results of logistic regression analysis using angle

| Variables | Odds ratio | 95% CI | *P*-value |
| --- | --- | --- | --- |
| Age (each 10-year increment) | 0.88 | 0.76−1.04 | 0.14 |
| Body mass index | 1.04 | 0.99−1.10 | 0.13 |
| Sex (female) | 1.35 | 0.85−2.15 | 0.20 |
| Patient category (surgical) | 0.71 | 0.45−1.12 | 0.14 |
| Experience of physician (non-resident) | 0.59 | 0.36−0.97 | 0.04 |
| Angle (each 10-degree increment) | 0.93 | 0.85−1.02 | 0.14 |
| Use of opioid | 0.52 | 0.30−0.91 | 0.02 |
| Use of vasopressor | 0.68 | 0.42−1.11 | 0.12 |

*Angle* was defined as the (a) the line between the lowest point of the serosal side of the greater curvature and the lower point of the caudal and serosal side of the pylorus, and (b) the horizontal line. Odds ratio >1.0 are associated with successful placement of enteral feeding tube. *CI* confidence interval.
