# Supplementary material for: Stomach position evaluated using computed tomography is related to successful post-pyloric enteral feeding tube placement in critically ill patients: a retrospective observational study
Source: J Intensive Care. 2023 May 30;11:25. doi: 10.1186/s40560-023-00673-4 (PMC10228095; doi:10.1186/s40560-023-00673-4)
Supplement: Supplementary file 5 — Additional file 5. Sensitivity analysis for primary outcome excluding experience of the physician. Sensitivity analysis of the primary outcome that did not include physician experience as a covariate. [file 40560_2023_673_MOESM5_ESM.docx]

**Additional file 5.** Sensitivity analysis for primary outcome excluding experience of physician

| Variables | Odds ratio | 95% CI | *P*-value |
| --- | --- | --- | --- |
| Age (each 10-year increment) | 0.89 | 0.77−1.02 | 0.08 |
| Body mass index | 1.03 | 0.98−1.07 | 0.27 |
| Sex (female) | 1.82 | 1.19−2.79 | 0.006 |
| Presence of intestinal peristaltic movement | 0.84 | 0.52−1.37 | 0.49 |
| Serum albumin level (each 1 mg/dL increment) | 0.81 | 0.26−2.57 | 0.73 |
| Diabetes mellitus | 1.02 | 0.63−1.67 | 0.92 |
| Hiatal hernia | 0.74 | 0.29−1.89 | 0.53 |
| Position of the stomach (caudal to L2-3) | 0.52 | 0.33−0.81 | 0.004 |

Odds ratio >1.0 are associated with successful placement of enteral feeding tube. *CI* confidence interval.
